# Editorial: Advances in retinal imaging biomarkers for cerebrovascular diseases

**DOI:** 10.3389/fnins.2024.1517573

**Published:** 2024-11-15

**Authors:** Youjie Wang, Rumei Lei, Le Cao, Hang Wang, Bo Wu, Shuai Jiang

**Affiliations:** Department of Neurology, West China Hospital, Sichuan University, Chengdu, China

**Keywords:** cerebrovascular disease, cerebral small vessel disease, neuro-ophthalmology, optical coherence tomography, retina

Over the past decade, ocular imaging strategies have significantly improved the detection of neurodegeneration and microvascular changes in a wide range of cerebrovascular diseases (Kashani et al., 2021). Advances in technologies such as fundus photography, optical coherence tomography (OCT), and OCT angiography (OCTA) have provided new insights into the disease mechanism of patients with stroke, internal carotid artery stenosis (ICAS), and dementia. Recently, multimodality ocular imaging approaches have expanded to systematically assess various ocular structures including the optic nerve head (ONH), retina, and choroid. Such multimodality imaging enhances the detection of neurodegeneration and microvascular changes, providing objective information on the disease which is useful for prognosis. Given its accessibility, ocular imaging could be integrated into neurology clinics, enabling direct evaluation of ocular tissues that reflect brain health. However, the exact contribution of these ocular metrics in various cerebrovascular diseases remains poorly understood. This editorial summarizes articles from the Research Topic “*Advances in retinal imaging biomarkers for cerebrovascular diseases*”, with the goal of enhancing our understanding of the value of retinal imaging in the diagnosis and treatment of cerebrovascular diseases.

Wang et al. systematically studied the relationship between retinal structure and vessel density changes and cerebral small vessel disease (CSVD) imaging markers, as well as the total CSVD burden, thus complementing existing research. Given the inherent presence of various CSVD imaging markers and retinal imaging markers, along with their rich spatial characteristics (e.g., different brain region CSVD imaging features and retinal features classified into four quadrants: superior, nasal, inferior, and temporal), numerous studies have explored the relationship between these factors (Lee et al., 2020; van de Kreeke et al., 2018). The authors reported a correlation between the vessel density of the superficial retinal capillary plexus (SRCP) and CSVD imaging manifestations, supporting small-vessel arteriosclerosis as a mechanism affecting both the retina and brain. This study also emphasized the need for robust and sensitive ophthalmic indicators, validated by longitudinal studies, to serve as non-invasive and economical markers reflecting CSVD.

Zhang et al. reviewed the evidence supporting the use of retinal imaging to diagnose various cerebrovascular diseases, including stroke, CSVD, vascular cognitive impairment, and subtypes of the former two conditions. From an anatomical and pathophysiological perspective, retinal imaging can be used for the early diagnosis and prevention of cerebral vascular diseases because the retina and optic nerve are connected to the central nervous system, while the retinal artery and vein are linked to the internal carotid artery system and internal jugular vein, respectively (Patton et al., 2005). Overall, a growing body of research has found that retinal microvasculature disruption, reduced retinal nerve fiber layer (RNFL) thickness and other static morphological metrics are indicative of cerebrovascular diseases. Additionally, measurements of dynamic blood flow conditions, such as blood flow velocity, might reflect the total burden of CSVD. However, one major challenge of using multimodal retinal imaging to detect cerebrovascular diseases in their early stages is that many retinal imaging features are difficult to correlate with specific cerebrovascular diseases due to the complex interplay of various pathological processes and the influence of confounding factors, such as hypertension and diabetes (Hughes et al., 2016).

The systematic review and meta-analysis conducted by Hou et al. highlight the value of OCT/OCTA as a tool that can quantitatively measure the structural and microvascular changes in the retina and choroid of internal carotid artery stenosis (ICAS) and provide a more nuanced understanding of ICAS's biological underpinnings. ICAS is well-established as a risk factor for cerebral ischemic events, contributing to 10–20% of strokes and transient ischemic attacks, and it can lead to cognitive impairment through mechanisms such as hypoperfusion, microembolization, and cerebrovascular reactivity (Gutierrez et al., 2022). An impaired blood flow in the internal carotid artery can influence blood flow in the ocular circulation since the ophthalmic artery, which is the first branch of the internal carotid artery, gives rise to the posterior ciliary artery and central retinal artery, resulting in ocular symptoms and signs on the ipsilateral side of the affected carotid artery including amaurosis fugax or ocular ischemic syndrome. The authors found that eyes on the ipsilateral side had reduced retinal ganglion cell thickness compared to controls. Nevertheless, there is no significant difference in the choroidal thickness. The study also observed a lower radial peripapillary capillary density around the optic nerve head in ICAS patients, suggesting microcirculatory impairment in the retina. This research is crucial for developing precision therapies targeting ICAS. For instance, retinal ganglion cells are significant neurons for retinal signaling and damage to their function or a reduction in their structural thickness can directly affect visual processing. In addition, these layers are affected by various cerebrovascular diseases, including stroke (Cuenca et al., 2020; Kingsbury et al., 2020).

This Research Topic of studies focuses on elucidating the impact of various cerebrovascular disorders on the eye by exploring their effects on the structure and microvasculature. Such research provides new perspectives for the diagnosis and treatment of various cerebrovascular diseases, allowing for more targeted approaches based on the specific characteristics of each condition. For example, while cerebral small vessel disease affects the total retinal thickness, retinal microvasculature, and choriocapillaris, ICAS primarily impacts the radial peripapillary capillary network (capillaries around the optic nerve head). Furthermore, the implications of these findings could extend beyond cerebrovascular diseases, as many neurological conditions, such as Alzheimer's disease (AD), share overlapping features with cerebrovascular disorders. Thus, insights from cerebrovascular research may benefit broader neurological studies. To deepen our understanding, a comprehensive framework integrating genetic risk factors, neuroimaging, and ocular imaging across hierarchical levels, along with longitudinal studies on the long-term effects of structural and microvascular changes in the eye, is necessary. In conclusion, this Research Topic has highlighted the diverse effects of cerebrovascular diseases on ocular health. A multidisciplinary approach to these conditions provides deeper insights into ocular changes, and we hope that this research will stimulate further exploration of the hierarchical impact of cerebrovascular diseases on the eye, paving the way for more effective and targeted diagnostic and therapeutic strategies.
